# Ballistic Performance of Guaruman Fiber Composites in Multilayered Armor System and as Single Target

**DOI:** 10.3390/polym13081203

**Published:** 2021-04-08

**Authors:** Raphael Henrique Morais Reis, Larissa Fernandes Nunes, Fernanda Santos da Luz, Verônica Scarpini Candido, Alisson Clay Rios da Silva, Sergio Neves Monteiro

**Affiliations:** 1 Department of Materials Science, Military Institute of Engineering—IME, Praça General Tibúrcio 80, Urca, Rio de Janeiro 22290-270, Brazil; raphaelreis@ime.eb.br (R.H.M.R.); larissafernandes@ime.eb.br (L.F.N.); fsl.santos@gmail.com (F.S.d.L.); 2 Engineering of Natural Resources of the Amazon Program, Federal University of Pará—UFPA, Rua Augusto Corrêa 01, Belém, Pará 66075-110, Brazil; scarpini@ufpa.br (V.S.C.); alissonrios@ufpa.br (A.C.R.d.S.)

**Keywords:** guaruman fiber, epoxy composite, ballistic performance, multilayered armor system, stand-alone test

## Abstract

Multilayered armor systems (MAS) with a front ceramic layer backed by a relatively unknown Amazonian guaruman fiber-reinforced (*Ischnosiphon koem*) epoxy composites, as second layer, were for the first time ballistic tested against the threat of 7.62 mm rifle ammunition. The amount of 30 vol% guaruman fibers was investigated in three distinct configurations: (i) continuous aligned, (ii) 0–90° cross-laid, and (iii) short-cut randomly dispersed. Additionally, single-target ballistic tests were also carried out in the best MAS-performed composite with cross-laid guaruman fibers against .22 caliber ammunition. The results disclosed that all composites as MAS second layer attended the US NIJ standard with corresponding penetration depth of (i) 32.9, (ii) 27.5, and (iii) 29.6 mm smaller than the lethal limit of 44 mm in a clay witness simulating a personal body. However, the continuous aligned guaruman fiber composite lost structural integrity by delamination after the 7.62 projectile impact. By contrast, the composite with cross-laid guaruman fibers kept its integrity for subsequent shootings as recommended by the standard. The single-target tests indicated a relatively higher limit velocity for .22 caliber projectile perforation, 255 m/s, and absorbed energy of 106 J for the cross-laid guaruman fibers, which are superior to corresponding results for other less known natural fiber epoxy composites.

## 1. Introduction

Multilayered armor systems (MASs) have been investigated and applied as inserted plates in vests for personal ballistic protection against the threat of high-velocity ammunition [1,2,3]. A typical MAS has a brittle front ceramic layer, which is hard enough to erode the projectile [4]. Once this projectile, such as NIJ level III 7.62 mm [5], strikes the front ceramic, an impact compressive shock wave travels through the material and is reflected as tensile wave that shatters the brittle ceramic, absorbing more than 50% of the impact energy [6]. A second layer is selected as a lighter material able to reflect the tensile wave and reduce the impact energy even more by capturing the shattered ceramic and projectile fragments. Synthetic laminate panel, as second layer, and ductile metallic sheet, as third layer, might complement the MAS. Its ballistic efficiency is evaluated by standing as target backed by clay witness simulating a human body. The NIJ standard [5] requires that the target should not only stop the projectile, but also prevent an indented depth of penetration, known as backface signature (BFS), in the clay witness not exceeding 44 mm.

According to Medvedovski [3], different ceramics are being used as the MAS front layer, mostly alumina (Al_2_O_3_) but also non-oxide compounds such as B_4_C, SiC, Si_3_N_4_, and AlN, among others. As for the MAS second layer, synthetic laminates based on aramid fiber, under the commercial trademarks of Kevlar^TM^ and Twaron^TM^, as well as ultra-high molecular weight polyethylene (UHMWPE), under the commercial trademarks of Dyneema^TM^ and Spectra. In the past, poly(p-phenylene-2,6-benzobisoxazole) (PBO), Zylon^TM^, was used in bulletproof vests [7]. In addition to experimental works, theoretical modeling contributes to understand the ballistic behavior of laminated fiber composites. Indeed, finite element numerical models and simulations were applied to assess the ballistic impact of Kevlar and glass fiber composites [8,9] as well as the residual velocity and damage extent [10]. In more fundamental analysis, fractal modeling investigated transport phenomena through fibrous media [11,12], which might simulate the complex impact energy propagation in fiber-reinforced composites.

**Figure 1:**
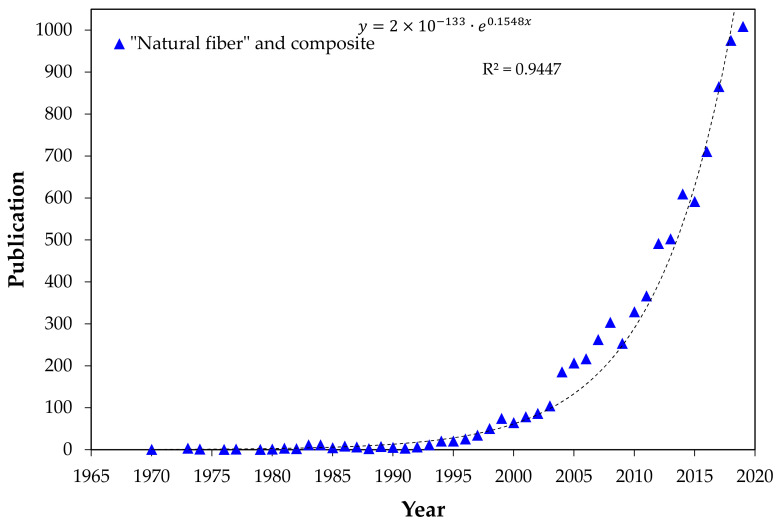
Publication by year for “Natural fiber and composite” according to the Scopus database [13].

Recently, more sustainable, low-density, and cost-effective natural lignocellulosic fiber (NLF)-reinforced polymer composites are being investigated as alternatives for synthetic laminates. Indeed, an exponential increase (Figure 1) in research works [13,14,15,16,17,18,19] on these NLF composites is motivating a surge in technological applications from civil construction panels to automobile parts [20,21,22]. Polymer composites reinforced with NLFs have also been successfully tested for their ballistic performance since 2007 [23]. Furthermore, recent review articles [24,25,26] reported an increasing tendency to use NLF-reinforced polymer composites for ballistic applications. In spite of being much weaker than synthetic aramid, UHMWPE, and PBO fibers, NLFs possess a similar capacity to dissipate the ballistic energy by capturing the fragments generated after projectile impact against the MAS front ceramic [1,7,27,28,29,30,31,32,33]. So far, the ballistic performance of these MASs was investigated with a second layer mostly of thermoset polymer matrix composites incorporated with well-known NLFs such as pineapple leaf [7], sisal [28,33], curaua [1,29], mallow [30], coir [31], and bamboo [32]. In particular, a less-known piassava fiber from Brazil [27] was recently found to provide efficient ballistic performance reinforcing an epoxy matrix composite as MAS second layer. This motivated the present work to investigate for the first time the ballistic performance of MAS with a second layer of guaruman fiber-reinforced epoxy composite. Additionally, single-target tests, technically known as standalone tests [34], were carried out in the most promising guaruman composite to evaluate the ballistic performance against medium velocity ammunition.

**Figure 2:**
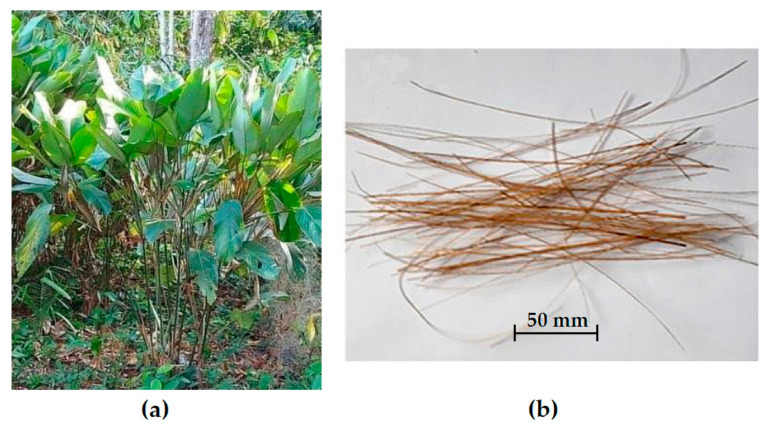
Guaruman: (**a**) plant and (**b**) fibers extracted from splinted stem (adapted from Reis et al. [35]).

The guaruman is an Amazonian shrub-like plant, scientifically known as *Ischnosiphon koem* (see Figure 2a), which has traditionally been used in the north of Brazil to fabricate simple items such as nets, rugs, ropes, and clothes. Local producers manually extract fibers (see Figure 2b) from splints mechanically cut from the guaruman stem [35].

Recently characterized guaruman fibers revealed properties (see Table 1) favorable to possible application of polymer composite reinforcement [35].

In Table 1, note that the density of guaruman fiber is among the lowest reported for natural lignocellulosic fibers. Moreover, the microfibrillar angle is coherent with a superior reported tensile strength [36]. These previous results constitute a relevant motivation for the use of guaruman fibers in innovative research issues related to ballistic armor for personal protection.

Considering numerous reported works using epoxy matrix composites reinforced with NLFs as well as natural fabric and bagasse [1,7,27,30,31,32,33,37,38,39,40,41], the present work evaluates the ballistic performance of MASs with Al_2_O_3_/Nb_2_O_5_ ceramic front layer followed by epoxy matrix composites reinforced with 30 vol% guaruman fiber, as second layer, and backed by a Kevlar^TM^ panel with 12 layers simulating a bulletproof vest-level IIIA. Ballistic tests are conducted with both high-speed 7.62 mm and medium speed .22 caliber ammunitions to assess the MAS BFS and standalone composites absorbed impact energy, respectively. Three distinct configurations of guaruman fibers embedded into the epoxy matrix are investigated.

## 2. Materials and Methods

### 2.1. Materials

**Figure 3:**
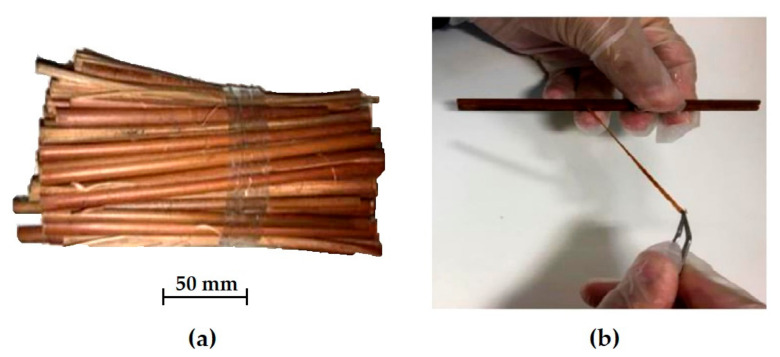
(**a**) As-purchased guaruman splints, and (**b**) manual separation of fiber from the splint (adapted from Reis et al. [35]).

Guaruman splints, mechanically cut from the stem, were purchased at local market in the city of Belem, Para, Brazil. From each splint, fibers were manually separated with a razor as illustrated in Figure 3. Extreme care was exercised in order to guarantee fiber extraction (see Figure 3b) with minimum damage. The razor is used to initiate a separation of each of the fibers bound together in the splint. Actually, natural fiber properties depend more on its individual diameter than the separation method either performed manually or industrially extracted [36].

The composite polymer matrix was a commercial epoxy resin diglycidyl ether of the bisphenol-A (DGEBA) hardened with triethylenetetramine (TETA) in stoichiometric ratio of 13 parts per 100 of resin. Both resin and hardener were fabricated by Dow Chemical and supplied by Epoxy Fiber, Brazil. As found in several publications on DGEBA/TETA epoxy matrix composites, the amount of 30 vol% natural fiber incorporation is the optimum for the most effective reinforcement [7,27,30,31,32,33,37,38,39,40,41,42,43,44]. Higher amounts make the fiber adhesion to the composite matrix difficult.

### 2.2. Composite Fabrication

**Figure 4:**
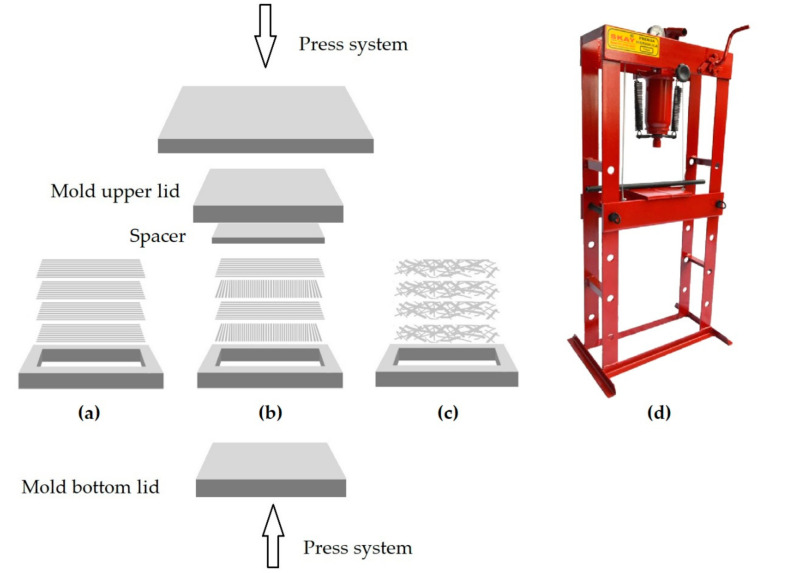
Schematic configurations of 30 vol% guaruman fibers in the mold: (**a**) continuous aligned, (**b**) 0–90° cross-laid, (**c**) short cut randomly dispersed, and (**d**) hydraulic press.

Guaruman fibers (see Figure 3b) were initially cut to 150 mm length, water cleaned, and dried in a stove at 80 °C for 24 h until their weight remained constant. A steel mold with inside dimensions of 150 × 120 × 12 mm^3^ was used to prepare composite plates by precisely hand lay-up 30.78 g of guaruman fibers, corresponding to 54 cm^3^ by the average density of 0.57 g/cm^3^ (Table 1). Separately, the fibers were laid in three distinct configurations schematically shown in Figure 4: (a) continuous aligned fibers along the mold greater 150 mm dimension, (b) 0–90° cross-laid long fibers, and (c) short-cut randomly dispersed fibers. In Figure 4b, the mold bottom and upper lids as well as the 2 mm thick spacer that limits, upon closing, the composite plate thickness to 10 mm are schematically shown.

For each configuration, after hand lay-up exactly 54 cm^3^ of guaruman fibers in the mold 180 cm^3^ open space, a 126 cm^3^ of DGEBA/TETA epoxy, still fluid, was poured. The mold was then closed and kept under pressure of 5 tons at room temperature (RT) for 24 h in a Skay hydraulic press (Brazil), illustrated in Figure 4d. Afterwards, the plate was unmolded and post-cured for one week at RT. Similar epoxy laminate plates reinforced with 30 vol% of continuous and aligned guaruman fibers [45] displayed significant tensile strength properties associated with good statistical repeatability, which supports the present investigation on ballistic performance.

### 2.3. Multilayered Armor Assembly

**Figure 5:**
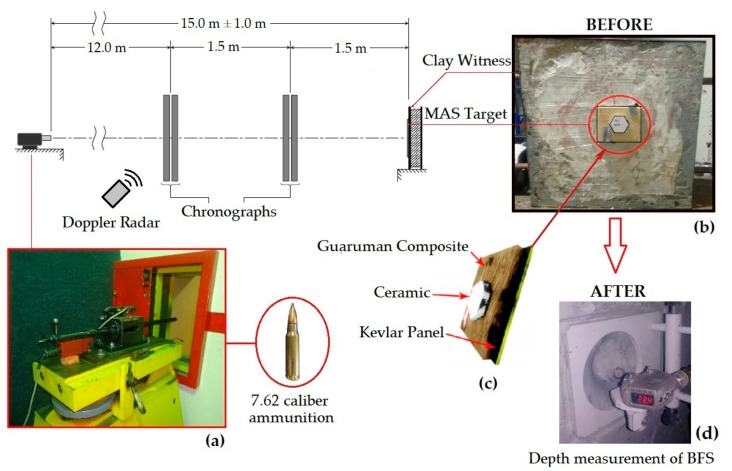
Schematic diagram of the setup arrangement at CAEx for MAS ballistic test (adapted from the work in [5]) with insets of (**a**) gun barrel and ammunition, (**b**) scheme of MAS target, (**c**) MAS target and clay witness block, and (**d**) laser measurement of BFS.

MAS target samples were mounted with a 10 mm thick front layer hexagonal Al_2_O_3_-4wt% Nb_2_O_5_ ceramic tile glued with polyurethane adhesive to the guaruman composite second layer and backed by a Kevlar^TM^ panel, as schematically shown in the inset of Figure 5c. The harder and more brittle front ceramic tile shatters the projectile and is microscopically fragmented, forming a cloud of particles that is captured by the guaruman composite and Kevlar back layers.

### 2.4. Ballistic Tests

Two distinct ballistic tests were conducted at Brazilian army facilities: (i) MASs with measured BFS of indented penetration in 3 samples for each guaruman fiber configuration and (ii) composite standalone with measurements of the after perforation residual velocity performed in 20 samples.

As for the MASs, a 7.62 × 51 mm^2^ caliber ammunition was used with a 9.7 g projectile and impact velocity of 838 ± 15 m/s, corresponding to an energy of ~3.4 kJ. These MASs ballistic tests were performed at the Army Assessment Center (CAEx), Rio de Janeiro, Brazil. Figure 5a–c schematically shows the standard CAEx ballistic setup for shooting high-velocity ammunition from a gun barrel. In this figure, behind the MAS a block of Roma-type clay witness is placed to record the projectile BFS, which is associated with the depth of penetration. The indentation of this depth of penetration in the clay witness was determined by means of a laser sensor model Q4X Banner (Figure 5d). The laser sensor was manually scanned to obtain the deepest penetration in the clay witness hole caused by the remaining cloud of fragments from the projectile impact against the MAS front ceramic. The deepest penetration measurement was considered the BFS. In order to evaluate the existence of difference between the BFS results, the statistical analysis of variance (ANOVA) was performed.

**Figure 6:**
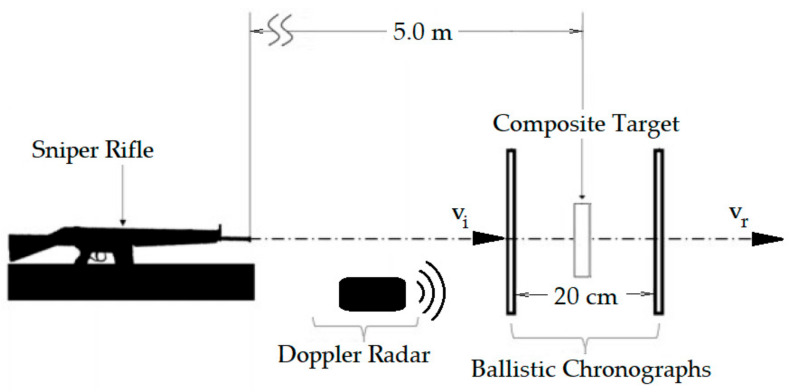
Schematic diagram of the setup arrangement at the Military Institute of Engineering (IME) for composite standalone ballistic test. Adapted from the work in [5].

As for the composite standalone ballistic test, a .22 caliber ammunition was used with 3.3 g of mass projectile and impact velocity of 284 ± 10 m/s, corresponding to an energy of ~0.13 kJ. These standalone tests were conducted at the Military Institute of Engineering (IME), Rio de Janeiro, Brazil. Figure 6 schematically shows the IME standalone test setup using a Gunpower SSS sniper rifle with weapon noise suppressor. The rifle was positioned 5 m away from the target, which consists of a composite plate fixed to a frame by a clamp. As indicated in Figure 6, two chronograph models (MK3 Air Chrony and Pal Chronos) were placed 10 cm before and 10 cm after the composite plate target, respectively, to measure the impact velocity ($v_{i}$) and the residual velocity ($v_{r}$). The composite absorbed impact energy (*E_abs_*) is calculated as

(1) $$E_{abs}=\frac{m_{p}\cdot \left(v_{i}{}^{2}-v_{r}{}^{2}\right)}{2}-E_{0}$$

where *m_p_* is the mass of the projectile and *E*_0_ is the energy dissipated by projectile only during its air flying trajectory.

In both different ballistic tests, MAS in Figure 5 and standalone in Figure 6, the projectile hit the target front surface in a 90° angle, characterizing a perpendicular impact recommended by the standard [34].

### 2.5. Scanning Electron Microscopy (SEM)

SEM analysis of the guaruman fiber-reinforced epoxy composite’s fractured surface, after the ballistic tests, was carried out in a model Quanta FEG 250 Fei microscope operating with secondary electrons between 5 and 15 kV. SEM composite samples were gold-sputtered before observation with secondary electrons.

## 3. Results and Discussion

Table 2 presents the BFS, associated with the depth of penetration measured by laser sensor, illustrated in Figure 5c, for MASs with second layer of epoxy composites reinforced with 30 vol% of guaruman fibers in the three distinct configurations indicated in Figure 4. These configurations are now abbreviated in Table 2 as continuous aligned (CA), cross-laid (CL), and short-cut (SC) fibers.

In Table 2, it is noteworthy that for all tested MASs there was no complete perforation of the target and the depth of penetration was less than 44 mm, which adheres to the NIJ standard for body protection [5]. Within the standard deviations, the BFS values in Table 2 are practically the same. These values are comparable to those (26.6 ± 2.0 mm) recently obtained for a continuous and aligned 30 vol% pineapple leaf fiber (PALF)-reinforced epoxy composite second layer in a similar MAS [7]. Table 3 shows the ANOVA parameters for the BFS values in Table 2. In this analysis, the degree of freedom (DF) corresponds to the minimum number of independent parameters. The total DF is n×k−1, where *n* is the number of treatments and *k* the number of samples. The residual DF is the difference between the total and treatment DF values. In Table 3, the calculated F_cal_ (3.8) is less than the critical F_crit_ (5.1). Therefore, the BFS averages in Table 2 are not significantly different with 95% level of confidence. Therefore, the way the fibers are arranged in the epoxy matrix does not change the ballistic armor efficiency in terms of indented penetration depth.

**Figure 7:**
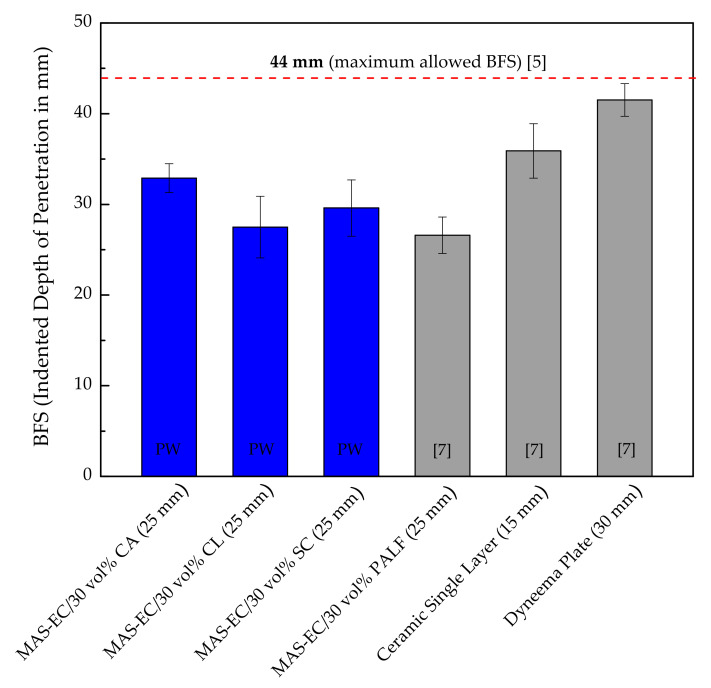
Bar graphs comparing MASs second layer with epoxy composites with 30 vol% of guaruman present work (PW) and 30 vol% PALF [7] as well as single ceramic and Dyneema^TM^ [7]; all backed by 5 mm thick Kevlar^TM^ panel. Total target thickness indicated in parentheses. References indicated inside the bars in brackets.

Figure 7 shows a graphical comparison between the present results of BFS in Table 2, including epoxy composites (EC) reinforced by 30 vol% guaruman fibers in different configurations (CA, CL, and SC), with that of similar EC with 30 vol% PALF [7], both as a 10 mm thick MAS second layer backed with 5 mm thick Kevlar^TM^ panel. In this figure, the BFS of a 10 mm thick single layer of the same Al_2_O_3_ 4 wt% Nb_2_O_5_ ceramic is also compared as well as a single 25 mm thick plate of Dyneema^TM^, both backed by 5 mm thick Kevlar^TM^ panel, reported by Luz et al. [7]. In particular, the lighter Dyneema/Kevlar^TM^ plate has a BFS value of 41.5 ± 1.8 mm, which is very close to the allowed standard limit of 44 mm [5]. The Dyneema^TM^ is a common hard armor plate used as an insert for ballistic vest protection against high-velocity ammunition by military servicemen in many countries, including Brazil. On the other hand, MASs’ front ceramic layer followed by either guaruman composites (present work) or PALF composite [7], both backed by Kevlar^TM^, although heavier, has significantly lower BFSs, which might allow further reduction in MAS thicknesses. In fact, the BFS value of 35.9 ± 3.0 mm (<44 mm) for the single ceramic/Kevlar^TM^ in Figure 7 indicates that the 10 mm thick front ceramic layer in both the present and [7] other research works can still be further reduced and, as such, decrease the MAS armor plate weight. Indeed, this would upset today’s main advantage of Dyneema^TM^ over MASs with a second layer including not only the present work and that in [7], but also those with a 5 mm thick third layer of aluminum alloy [1,28,29,30,31,32,33,46,47,48,49]. Note that among these MASs with three layers (ceramic + NLF composite + Al alloy), some also reported that using Kevlar^TM^ as a second layer [41], the BFS was found to be 21–23 mm. In other words, Kevlar^TM^ in MAS could also compete against Dyneema^TM^, single plate.

**Figure 8:**
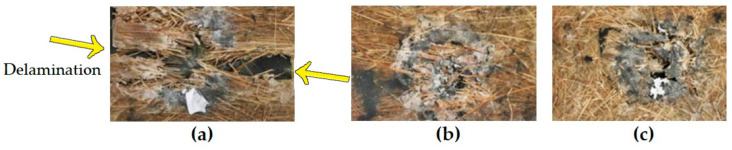
General macroscopic aspect after ballistic test of MAS targets with second layer of epoxy composites reinforced with 30 vol% of guaruman fibers: (**a**) continuous aligned, (**b**) cross-laid, and (**c**) short-cut randomly dispersed; all backed by 5 mm thick Kevlar^TM^ panel.

Figure 8 shows the final aspect of MAS targets after the 7.62 mm projectile impact, which completely destroyed the hexagonal ceramic tile; some white fragments still remain attached. In this figure, note that the target with continuous aligned long guaruman fibers (CA) (see Figure 8a) suffered delamination, indicated by arrows, which will not protect against subsequent shootings. By contrast, targets with cross-laid (CL) (see Figure 8b) and short-cut dispersed (SC) (see Figure 8c) guaruman fibers will stand other shooting as required by the NIJ standard [34] for personal protection. This standard requires that a body armor should withstand six consecutive shootings, all with BFS lower than 44 mm in a clay witness placed behind the armor. If delamination occurred in the first shooting, as in Figure 8a, the subsequent shootings have a chance to go through the open delaminated space and cause a complete perforation in the clay witness. In the present work, delamination occurred in the aligned fiber (CA) because the composite has only major resistance along 1D. Cross-laid (CL) or short-cut dispersed (SC) fibers provided 2D resistance, which prevents delamination, as shown in Figure 8b,c.

Table 4 presents the main parameters and results obtained for the single-target standalone ballistic tests with .22 caliber ammunition for the epoxy matrix epoxy composite reinforced with 30 vol% of guaruman fibers in the configuration of cross-laid long fibers. The selection of this composite for standalone test was due to the best BFS average in MAS ballistic test (see Table 2) as well as a composite that kept its integrity (see Figure 8b) after shooting. In Table 4, the limit velocity ($v_{L}$), corresponding to the highest projectile velocity for which the composite is not perforated is also presented. According to Morye et al. [50].

(2) $$v_{L}=\sqrt{\frac{2\cdot E_{abs}}{m}}$$

where *E_abs_* is the composite absorbed ballistic impact energy from the projectile with mass *m*. Based on the relatively large number of (20) samples tested, it was possible to perform the Weibull statistical analysis to determine the degree of dispersion and level of precision associated with the .22 caliber ammunition ballistic results. Both dispersion and precision in ballistic experimental results are of fundamental relevance for comparison with our ongoing finite element modeling work following recent papers on this subject [8,9,10]. Weibull parameters *β* and *θ*, as well as the correlation coefficient R^2^, were obtained from the frequency distribution function for five tests.

(3) $$f\left(x\right)=\exp\left[-{\left(\frac{x}{\theta }\right)}^{\beta }\right]$$

where *β* is the Weibull modulus, which indicates a smaller dispersion of test results, the higher is its value.

The results in Table 4 revealed that the 10 mm thick epoxy composite reinforced with 30 vol% of guaruman cross-laid fibers can resist a limit velocity (254.7 m/s) without perforation in association with a greater statistical precision (R^2^ = 0.96). Moreover, with the same precision, the guaruman composite plate absorbs an average energy of 105.5 J from the impact of a .22 caliber projectile, which is superior to those of other less known NLF epoxy composites compared in Table 5.

**Figure 9:**
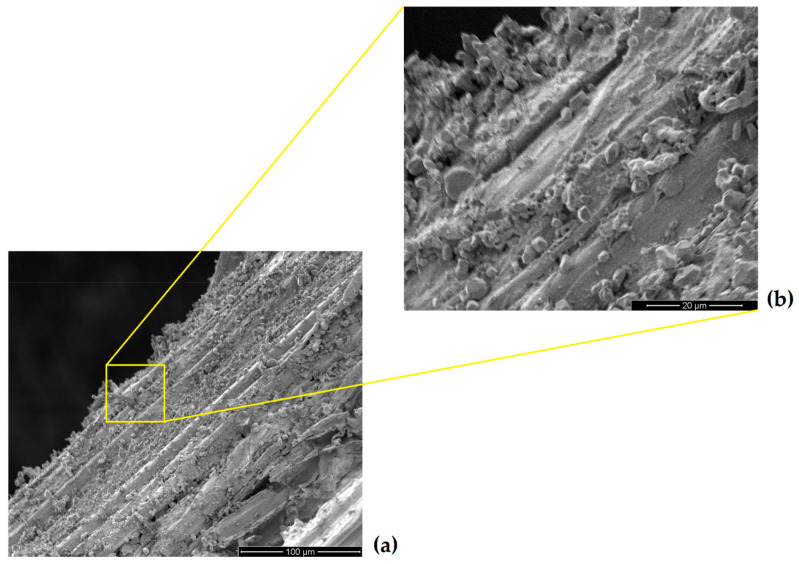
SEM fractographs of the epoxy composite with 30 vol% guaruman cross-laid fibers as MAS second layer after ballistic test: (**a**) fiber covered with ceramic fragments from the shattered front layer and (**b**) high magnification of ceramic particles covering the fiber.

SEM fractographs of the cross-laid guaruman fiber composite after the ballistic test as MAS second layer are shown in Figure 9. As in other ballistic results of MASs with NLF composites [1,7,28,29,30,31,32,33,37,38,39,40,41,44,46,47,48,49,50], the main mechanism of energy absorption by the second layer is the capture of fragments resulting from the ceramic shattering, see Figure 9a. With higher magnification, small ceramic particles are revealed in Figure 9b. This capture of fragments mechanism associated with van der Waals forces and electrostatic attraction was first disclosed by Monteiro et al. [6] in the aramid fibers of Kevlar^TM^ as MAS second layer. The 7.62 mm impact against the front ceramic (see Figure 5b,c) causes a compression wave to advance into the target and to reflect at the guaruman composite second layer as a tensile wave. The brittle ceramic is then shattered by the reflected tensile wave absorbing more than 50% of the impact energy [6]. A cloud of both ceramic and bullet fragments travels through the composite plate and is partially captured by the guaruman fibers (see Figure 9) without damage to the fiber. However, macroscopic fiber/epoxy delamination (see Figure 8a) occurs in the case of CA composites. This mechanism of fragment capture in a MAS with natural fiber composite as second layer dissipates more energy than a same thickness Kevlar [1]. Even though energy dissipation measurements are not yet available for guaruman fiber composites, we assume that they might be comparable to other natural fiber composites and more effective than Kevlar as MAS second layer. The remaining impact energy going through the guaruman composite and the Kevlar panel (see Figure 5c) causes the BFS illustrated in Figure 5d.

**Figure 10:**
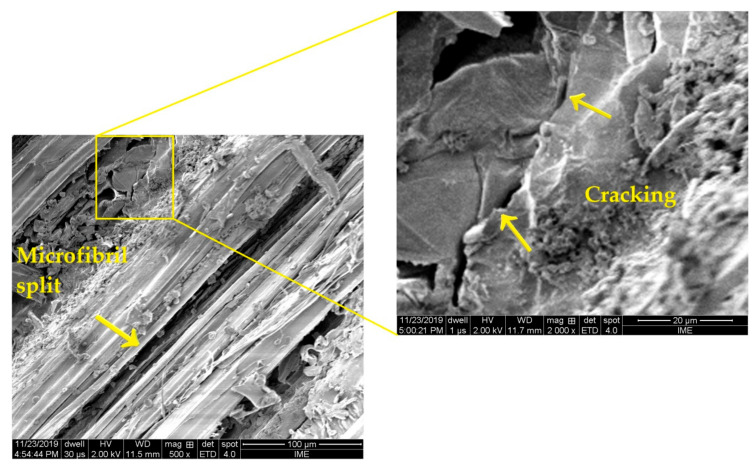
SEM fractograph of the epoxy composite with 30 vol% guaruman cross-laid fibers after standalone ballistic test.

Figure 10 shows SEM images of the fracture surface of epoxy composite reinforced with 30 vol% of guaruman cross-laid fibers after standalone ballistic test. Different than Figure 9, no ceramic fragments exist in this case. The main mechanisms of .22 caliber projectile impact absorption were found to be microfibril split of guaruman fibers, clearly seen in this figure, as well as cracking of the epoxy matrix pointed in the inset. The relatively high strength of the guaruman fiber (614 MPa in Table 1) prevents its rupture and contributes to keeping the integrity of the CL guaruman fiber composite, despite cracking of the epoxy matrix. As in the case of MAS against 7.62 mm ammunition (see Figure 8), the integrity of the CL guaruman fiber composites against subsequent shooting in the case of .22 caliber ammunition is an important requirement for personal ballistic armor.

## 4. Conclusions

- Ballistic tests were for the first time performed in epoxy composites reinforced with a less-known guaruman fiber from the Brazilian Amazon. Both tests using 30 vol% guaruman composites, either in a multilayered armor system (MAS) against 7.62 mm ammunition or as standalone target against .22 mm caliber ammunition, displayed promising results for personal protection.
- In particular, the guaruman long fibers in the 0–90° cross-laid configuration inside the epoxy matrix contributed, as MAS second layer, to a backface signature in clay witness of 27.5 mm, which is significantly lower than the limit of 44 mm required by the NIJ for lethal trauma. Additionally, contrary to composite with continuous aligned guaruman fiber, the cross-laid fibers composite after the ballistic test kept its integrity for subsequent shootings as recommended by the standard.
- The standalone tests revealed a limit velocity for projectile perforation of 255 m/s and absorbed ballistic energy of 106 J associated with the best composite with cross-laid guaruman fibers, which are superior to corresponding results for other less known natural fiber epoxy composites.
- Scanning electron microscopy (SEM) observations of the guaruman cross-laid fiber composites indicated, as expected, a main mechanism of energy absorption associated with the capture of fragments from the shattered ceramic MAS front layer. On the other hand, ballistic energy absorption mechanisms of epoxy matrix cracking and guaruman fiber split into microfibrils were found for the standalone tests.
- Ballistic tests of the cross-laid guaruman fiber-reinforced epoxy composite revealed for the first time a performance favorable to application not only as MAS second layer against high-velocity rifle bullets, but also single plate protecting against medium-velocity pistol bullets. Future research on ballistic numerical modeling is underway to confirm these experimental results.

## Figures and Tables

**Table 1:** Properties reported for guaruman fiber [35].

Density by Mass/Volume (g/cm^3^)	Density by Archimedes Method (g/cm^3^)	Microfibrillar Angle (degree)	Crystallinity Index (%)	Preliminary Tensile Strength (MPa)
0.50 ± 0.17	0.64 ± 0.19	7.8 ± 0.3	60-67	614 ± 21

**Table 2:** BFS from ballistic tests in MASs with second layer of 30 vol% guaruman fibers epoxy composites.

MAS with Second Layer Composites with 30 vol% of Guaruman Fibers	Continuous Aligned (CA)	Cross-Laid (CL)	Short-Cut (SC)
**Depth of penetration BFS (mm)**	32.9 ± 1.6	27.5 ± 3.4	29.6 ± 3.1

**Table 3:** Analysis of variance (ANOVA) applied to the BFS results of MASs with second layer of 30 vol% guaruman fibers epoxy composites, comparing distinct fibers configurations.

Source Variation Causes	Sum of Squares	Degrees of Freedom (DF)	Mean of Squares	F_cal_	F_crit_
Treatment	45	2	22	3.8	5.1
Residual	48	6	8		
Total	93	8			

**Table 4:** Standalone ballistic test results and Weibull parameter for the epoxy composite reinforced with 30 vol% of guaruman fibers in cross-laid configuration.

Parameter	Value	Weibull Parameter		
$v_{i}$ (m/s)	285.8 ± 5.3	*β*	*θ*	R^2^
$v_{r}$ (m/s)	5.4 ± 0.1			
*E_abs_* (J)	105.5 ± 10.6	10.8	110	0.96
$v_{L}$ (m/s)	254.7 ± 12.8	21.7	261	0.96

Note: mass of projectile is 3.31 ± 0.04 g and *E*_0_ = 2.69 ± 1.71 J.

**Table 5:** Absorbed energy and limit velocity obtained in .22 caliber standalone ballistic tests comparing the present result and those of other DGEBA/TETA epoxy composites reinforced with less known natural fibers.

30 Vol% Natural Fiber Reinforcing DGEBA/TETA Epoxy Composite	Absorbed Energy E_abs_ (J)	Limit Velocity $v_{L}\;(m/s)$	Reference
30 vol% Guaruman (*Ischnosiphon koem*)	105.5 ± 10.6	254.7 ± 12.8	PW
30 vol% Sedge (*Cyperus malaccensis*)	74.0 ± 2.5	212.5 ± 15.2	[51]
20 vol% Tucum (*Astrocaryum vulgare*)	84.0 ± 8.4	226.7 ± 16.1	[52]
40 vol% Tucum (*Astrocaryum vulgare*)	69.6 ± 9.1	204.4 ± 14.3	[52]
30 vol% Carnauba (*Copernicia prunifera*)	63.9 ± 4.6	194.1 ± 7.1	PC *
30 vol% Caranan (*Mauritiella armata*)	48.2 ± 8.3	186.0 ± 11.9	PC **

PW: Present work. PC *: Private communication; unpublished work of Junio, R.F.P. (raivsjfelipe@ime.eb.br). PC **: Private communication; unpublished work of Souza, A.T. (andressa.souza@ime.eb.br).

## Data Availability

Not applicable.

## References

[1] Monteiro S.N., Louro L.H.L., Trindade W., Elias C.N., Ferreira C.L., Lima E.S., Weber R.P., Suarez J.C.M., Figueiredo A.B.-H.S., Pinheiro W.A. (2015). Natural curaua fiber-reinforced composites in multilayered ballistic armor. Metall. Mater. Trans. A.

[2] Tasdemirci A., Tunusoglu G., Güden M. (2012). The effect of the interlayer on the ballistic performance of ceramic/composite armors: Experimental and numerical study. Int. J. Impact Eng..

[3] Medvedovski E. (2006). Lightweight ceramic composite armour system. Adv. Appl. Ceram..

[4] Medvedovski E. (2010). Ballistic performance of armour ceramics: Influence of design and structure. Part 1. Ceram. Int..

[5] National Criminal Justice Reference Service, US Department of Justice, & National Institute of Justice (2000). NIJ 0101.04. Ballistic Resistance of Body Armor. https://nij.ojp.gov/library/publications/ballistic-resistance-personal-body-armor-nij-standard-010104.

[6] Monteiro S.N., Lima E.P., Louro L.H.L., Silva L.C., Drelich J.W. (2014). Unlocking function of aramid fibers in multilayered ballistic armor. Metall. Mettal. Mater. Trans. A.

[7] da Luz F.S., da Costa Garcia Filho F., Oliveira M.S., Nascimento L.F.C., Monteiro S.N. (2020). Composites with Natural Fibers and Conventional Materials Applied in a Hard Armor: A Comparison. Polymers.

[8] Dewangan M.K., Panigrahi S.K. (2021). Finite element analysis of hybrid 3D orthogonal woven composite subjected to ballistic impact with multi-scale modeling. Polym. Adv. Technol..

[9] Scazzosi R., Manes A., Petrone G., Giglio M. (2018). Two different modeling approaches for fabric composites subjected to ballistic impact. IOP Conf. Ser. Mater. Sci. Eng..

[10] Key C.T., Alexander C.S. (2018). Experimental testing and numerical modeling of ballistic impact on S-2 glass/SC15 composites. J. Dyn. Behav. Mater..

[11] Xiao B., Huang Q., Chen H., Chen X., Long G. (2021). A fractal model for capillary flow through a single tortuous capillary with roughened surfaces in fibrous porous media. Fractals.

[12] Xiao B., Zhang Y., Wang Y., Jiang G., Liang M., Chen X., Long G. (2019). A fractal model for Kozeny–Carman constant and dimensionless permeability of fibrous porous media with roughened surfaces. Fractals.

[13] Luz F.S., da Costa Garcia Filho F., del-Río M.T.G., Nascimento L.F.C., Pinheiro W.A., Monteiro S.N. (2020). Graphene-Incorporated Natural Fiber Polymer Composites: A First Overview. Polymers.

[14] Zhang Z., Cai S., Li Y., Wang Z., Long Y., Yu T., Shen Y. (2020). High performances of plant fiber reinforced composites—A new insight from hierarchical microstructures. Compos. Sci. Technol..

[15] Sanjay M.R., Madhu P., Jawaid M., Senthamarakannan P., Senthil S., Pradeep S. (2018). Characterization and properties of natural fiber polymer composites: A comprehensive review. J. Clean. Prod..

[16] Pickering K.L., Efendy M.G.A., Le T.M. (2016). A review of recent developments in natural fiber composites and their mechanical performance. Compos. Part A.

[17] Güven O., Monteiro S.N., Moura E.A.B., Drelich J.W. (2016). Re-Emerging Field of Lignocellulosic Fiber-Polymer Composites and Ionizing Radiation Technology in their Formulation. Polym. Rev..

[18] Faruk O., Bledzki A.K., Fink H.-P., Sain M. (2012). Biocomposites reinforced with natural fibers: 2000–2010. Prog. Polym. Sci..

[19] Monteiro S.N., Lopes F.P.D., Ferreira A.S., Nascimento D.C.O. (2009). Natural-fiber polymer-matrix composites: Cheaper, tougher, and environmentally friendly. JOM.

[20] Kumar R., Ul-Haq M.I., Raina A., Anand A. (2019). Industrial applications of natural fibre-reinforced polymer composites–challenges and opportunities. Int. J. Sustain. Eng..

[21] Dunne R., Desai D., Sadiku R., Jayaramudu J. (2016). A review of natural fibres, their sustainability and automotive applications. J. Reinf. Plast. Compos..

[22] Thomas N., Paul S.A., Pothan L.A., Deepa B., Kalia S., Kaith B.S., Kaur I. (2011). Natural fibers: Structure, properties and applications. Cellulose Fibers: Bio-and Nano-Polymer Composites.

[23] Wambua P., Vangrimde B., Lomov S., Verpoest I. (2007). The response of natural fibre composites to ballistic impact by fragment simulating projectiles. Compos. Struct..

[24] Nurazzi N.M., Asyraf M.R.M., Khalina A., Abdullah N., Aisyah H.A., Rafiqah S.A., Sabaruddin F.A., Kamarudin S.H., Norrrahim M.N.F., Ilyas R.A. (2021). A Review on Natural Fiber Reinforced Polymer Composite for Bullet Proof and Ballistic Applications. Polymers.

[25] Nayak S.Y., Sultan M.T.H., Shenoy S.B., Kini C.R., Samant R., Shah A.U.M., Amuthakkannan P. (2020). Potential of Natural Fibers in Composites for Ballistic Applications—A Review. J. Nat. Fibers.

[26] Benzait S., Trabzon L. (2018). A review of recent research on materials used in polymer–matrix composites for body armor application. J. Compos. Mater..

[27] Garcia Filho F.C., Monteiro S.N. (2019). Piassava fiber as an epoxy matrix composite reinforcement for ballistic armor applications. JOM.

[28] Braga F.O., Bolzan L.T., Ramos F.J.H.T.V., Monteiro S.N., Lima E.P. (2018). Ballistic Efficiency of Multilayered Armor Systems with Sisal Fiber Polyester Composites. Mater. Res. Ibero Am. J..

[29] Monteiro S.N., Braga F.O., Lima E.P., Louro L.H.L., Drelich J.W. (2017). Promising curaua fiber-reinforced polyester composite for high-impact ballistic multilayered armor. Polym. Eng. Sci..

[30] Nascimento L.F.C., Louro L.H.L., Monteiro S.N., Lima E.P., Luz F.S. (2017). Mallow Fiber-Reinforced Epoxy Composites in Multilayered Armor for Personal Ballistic Protection. JOM.

[31] Luz F.S., Monteiro S.N., Lima E.S., Lima Júnior E.P. (2017). Ballistic application of coir fiber reinforced epoxy composite in multilayered armor. Mater. Res..

[32] Cruz R.B., Lima E.P., Monteiro S.N., Louro L.H.L. (2015). Giant bamboo fiber reinforced epoxy composite in multilayered ballistic armor. Mater. Res..

[33] Rohen L.A., Margem F.M., Monteiro S.N., Vieira C.M.F., Araújo B.M., Lima E.S. (2015). Ballistic efficiency of an individual epoxy composite reinforced with sisal fibers in multilayered armor. Mater. Res..

[34] National Criminal Justice Reference Service, US Department of Justice, & National Institute of Justice (2008). NIJ 0101.06. Ballistic Resistance of Body Armor. https://nij.ojp.gov/library/publications/ballistic-resistance-body-armor-nij-standard-010106.

[35] Reis R.H.M., Nunes L.F., Oliveira M.S., de Veiga Junior V.F., Garcia Filho F.D.C., Pinheiro M.A., Silva A.C.R., Candido V.S., Monteiro S.N. (2020). Guaruman fiber: Another possible reinforcement in composites. J. Mater. Res. Technol..

[36] Monteiro S.N., Lopes F.P.D., Barbosa A.P., Bevitori A.B., Da Silva I.L.A., Da Costa L.L. (2011). Natural Lignocellulosic Fibers as Engineering Materials—An Overview. Metall. Mater. Trans. A.

[37] Oliveira M.S., Garcia Filho F.C., Pereira A.C., Nunes L.F., Luz F.S., Braga F.O., Monteiro S.N. (2019). Ballistic performance and statistical evaluation of multilayered armor with epoxy-fique fabric composites using the Weibull analysis. J. Mater. Res. Technol..

[38] Braga F.O., Bolzan L.T., Luz F.S., Lopes P.H.L.M., Lima E.P., Monteiro S.N. (2017). High energy ballistic and fracture comparison between multilayered armor systems using non-woven curaua fabric composites and aramid laminates. J. Mater. Res. Technol..

[39] Monteiro S.N., Candido V.S., Braga F.O., Bolzan L.T., Weber R.P., Drelich J.W. (2016). Sugarcane bagasse waste in composites for multilayered armor. Eur. Polym. J..

[40] Monteiro S.N., Milanezi T.L., Louro L.H.L., Lima E.P., Braga F.O., Gomes A.V., Drelicch J.W. (2016). Novel ballistic ramie fabric composite competing with Kevlar^TM^ fabric in multilayered armor. Mater. Des..

[41] Luz F.S., Lima E.P., Louro L.H.L., Monteiro S.N. (2015). Ballistic test of multilayered armor with intermediate epoxy composite reinforced with jute fabric. Mater. Res..

[42] Garcia Filho F.C., Luz F.S., Oliveira M.S., Pereira A.C., Costa U.O., Monteiro S.N. (2020). Thermal behavior of graphene oxide-coated piassava fiber and their epoxy composites. J. Mater. Res. Technol..

[43] Luz F.S., Monteiro S.N., Tommasini F.J. (2018). Evaluation of dynamic mechanical properties of PALF and coir fiber reinforcing epoxy composites. Mater. Res..

[44] Costa U.O., Nascimento L.F.C., Garcia J.M., Monteiro S.N., Luz F.S., Pinheiro W.A., Garcia Filho F.C. (2019). Effect of graphene oxide coating on natural fiber composite for multilayered ballistic armor. Polymers.

[45] Pinheiro M.A., Gomes L.G., Silva A.C.R.D., Candido V.S., Reis R.H.M., Monteiro S.N. (2019). Guaruman: A Natural Amazonian Fiber with Potential for Polymer Composite Reinforcement. Mater. Res..

[46] Monteiro S.N., Salgado de Assis F., Ferreira C.L., Tonini Simonassi N., Pondé Weber R., Souza Oliveira M., Colorado H.A., Camposo Pereira A. (2018). Fique Fabric: A Promising Reinforcement for Polymer Composites. Polymers.

[47] Luz F.S., Ramos F.J.H.T.V., Nascimento L.F.C., Figueiredo A.B.H.S., Monteiro S.N. (2018). Critical length and interfacial strength of PALF and coir fiber incorporated in epoxy resin matrix. J. Mater. Res. Technol..

[48] Assis F.S., Pereira A.C., Garcia Filho F.C., Lima E.P., Monteiro S.N., Weber R.P. (2018). Performance of jute non-woven mat reinforced polyester matrix composite in multilayered armor. J. Mater. Res. Technol..

[49] Monteiro S.N., Pereira A.C., Ferreira C., Lima E.P. (2018). Performance of Plain Woven Jute Fabric-Reinforced Polyester Matrix Composite in Multilayered Ballistic System. Polymers.

[50] Morye S.S., Hine P.J., Duckett R.A., Carr D.J., Ward I.M. (2000). Modelling of the energy absorption by polymer composites upon ballistic impact. Compos. Sci. Technol..

[51] Mendonça Neuba L., Pereira Junio R.F., Ribeiro M.P., Souza A.T., de Sousa Lima E., Garcia Filho F.d.C., Figueiredo A.B.-H.S., Braga F.d.O., Azevedo A.R.G.d., Monteiro S.N. (2020). Promising Mechanical, Thermal, and Ballistic Properties of Novel Epoxy Composites Reinforced with *Cyperus malaccensis* Sedge Fiber. Polymers.

[52] Oliveira M.S., Luz F.S., Souza A.T., Demosthenes L.C.C., Pereira A.C., Garcia Filho F.C., Braga F.O., Figueiredo A.B.S., Monteiro S.N. (2020). Tucum Fiber from Amazon Astrocaryum vulgare Palm Tree: Novel Reinforcement for Polymer Composites. Polymers.

